# Improving organ dose sparing in left‐sided breast cancer with yaw‐limited volumetric modulated arc therapy: A dosimetric comparison to conventional and intensity modulated radiation therapy approaches

**DOI:** 10.1002/acm2.70041

**Published:** 2025-02-28

**Authors:** Gerhard Pollul, Sascha Grossmann, Heiko Karle, Tilman Bostel, Heinz Schmidberger

**Affiliations:** ^1^ Department of Radiation Oncology University Medical Center of the Johannes Gutenberg University Mainz Germany

**Keywords:** DIBH, left‐sided breast irradiation, partial VMAT, steep dose fall‐off, VMAT

## Abstract

**Background:**

To assess the dose‐sparing capabilities of a yaw‐limited volumetric modulated arc therapy (YL_VMAT) beam setup for adjacent organs at risk (OAR) in comparison with 3D‐conventional radiation therapy (3D‐CRT), intensity‐modulated radiation therapy (IMRT) and conventional VMAT for radiation therapy in left‐sided breast cancer patients.

**Methods:**

In total, 80 treatment plans for 20 patients, of which 10 patients underwent CT‐scans in deep inspiration breath‐hold (DIBH) and 10 patients in free‐breathing (FB) technique. Besides generally tangential‐weighted static and IMRT beams, VMAT treatment plans with approximately 270° arc length have been compared and analyzed to a multi‐field, yaw‐adapted, unconventional partial VMAT technique retrospectively. The prescription dose was set to 40.05 Gy in 15 fractions.

**Results:**

We achieved a more pronounced steeper dose falloff directed from the thoracic wall to the adjacent lung tissue resulting in a significantly better ipsilateral lung and considerably cardiac dose sparing using the YL_VMAT method in general. Compared with standard techniques (IMRT, VMAT, 3D‐CRT), YL‐VMAT in combination with DIBH can achieve lower mean doses for the heart (1.05 Gy vs. 1.73 Gy, 2.16 Gy and 1.44 Gy), the left anterior descending (LAD) artery (3.68 Gy vs. 6.53 Gy, 5.13 Gy and 8.64 Gy) and the left lung (3.59 Gy vs. 5.39 Gy, 4.79 Gy and 5.87 Gy), respectively. Also with FB, the corresponding mean doses for the left lung and cardiac structures were lower with the YL‐VMAT method than with IMRT (heart: 1.70 Gy vs. 2.44 Gy; LAD: 6.50 Gy vs. 11.97 Gy; left lung: 3.10 Gy vs. 4.72 Gy), VMAT (heart: 1.70 Gy vs. 2.52 Gy; LAD: 6.50 Gy vs. 9.06 Gy; left lung: 3.10 Gy vs. 4.46 Gy) and 3D‐CRT (heart: 1.70 Gy vs. 2.78 Gy; LAD: 6.50 Gy vs. 15.09 Gy; left lung: 3.10 Gy vs. 5.77 Gy). In addition, we found out superiority of YL_VMAT for the V5, V10, and V20 Gy to the left lung. For DIBH and FB, all differences for the left lung were significant, with *p* < 0.05.

**Conclusions:**

With the YL_VMAT technique, dose exposures to radiosensitive OARs like the lung, heart and LAD artery can be reduced considerably to very low values in comparison to already established planning methods. The benefits must be weighed against the potential risks induced by an increased dose exposure to the contralateral breast.

## 1 BACKGROUND

Breast cancer is the most common malignancy in women, accounting for approximately 25% of all malignancies.^1^ One in eight women will develop a breast cancer in the course of her life, with the predilection age being between 40 and 70 years.^2^ Left‐sided breast cancer is occurring slightly more frequently than right side. Additionally left sided breast cancer is associated with an increased aggressive biology and a worse outcome.^3^ After breast‐conserving surgery standard adjuvant treatment approach includes radiotherapy of the whole breast with or without boost.^4^ To achieve a sufficient dose coverage of the tumor affected breast, one established standard treatment design consist of tangential beams calculated forward with 3D‐CRT.^5^, ^6^ In recent years, inverse planning techniques like intensity modulated radiotherapy (IMRT) or volumetric modulated arc therapy (VMAT) with different beam arrangements have become established for radiation treatment of breast cancer.^7^, ^8^, ^9^, ^10^, ^11^ The usage of such methods can lead to a reduced dose for the adjacent OARs, such as the heart and the ipsilateral lung.^7^, ^12^, ^13^, ^14^, ^15^ Furthermore, patients treated in deep inspiration breath‐hold (DIBH) mode, benefit from a lower heart dose compared to free‐breathing (FB) mode.^16^, ^17^, ^18^, ^19^, ^20^ However, a respiratory management system is not available at all facilities and is more time‐consuming.

Dose‐sparing capabilities of the OARs or achieved dose gradients around the PTV are not only depending on the choice of the treatment method or breathing protocol, but also are a result of the beam‐size and MLC physical design. Depending on the field size, the transmission factor for VARIANs “Millennium 120 leaf MLC” for 6 MV FF ranges from about 1.36% to 2.45% according to literatures.^21^, ^22^, ^23^, ^24^ More radiation caused by MLC transmission can lead to an increased out‐of‐field dose.^25^ In addition, the inter‐leaf leakage in between two leaves is about 2%–3.5%.^26^, ^27^ Thus, one approach to reduce leakage radiation in OAR overlapping sections is the collimation of the MLC, achieved by yaw‐limited narrow field‐sizes. The aim of this study is to demonstrate the benefits of a partial yaw‐limited VMAT (YL_VMAT) beam setup compared to the usual established setup designs.

## 2 PATIENTS AND METHODS

### 2.1 Patients and contouring

This plan comparison study included 20 female patients with an average age of 47 years (range 32–61 years). All patients suffered from left‐sided invasive breast cancer and received adjuvant radiotherapy after breast‐conserving surgery. The left‐sided breast tissue was defined as the clinical target volume (CTV). The planning target volume (PTV) was generated by expanding the CTV by 5 mm radially and 10 mm craniocaudally, and then the PTV was cropped by 3 mm from the skin. The study patients had a mean breast size of 549 cc (range 274–833 cc). All OARs were contoured by using artificial intelligence (AI) based contouring software (Contour+, MVision AI, Helsinki, Finland).

### 2.2 Patients positioning

All patients were CT‐scanned in a supine position lying on a “Kombiboard” (UNGER Medizintechnik, Mühlheim‐Kärlich, Germany) to place their arms above the head and wedging the upper part of the body. Ten patients (Group 1) underwent CT‐scans in DIBH and 10 patients (Group 2) in FB procedure. Breathing amplitude was triggered and observed via the Real‐Time‐Position Management System (RPM, Varian Medical Systems, Palo Alto, USA) for the DIBH protocol. The slice thickness was 2 mm and pixel size was 0.9–1.1 mm, depending on the selected field of view.

### 2.3 Dose calculation

Treatment plan creation has been done with the treatment planning system (TPS) “Eclipse” from Varian (Varian Medical Systems, Palo Alto, CA, USA), and calculation has been performed with the “Anisotropic Analytical Algorithm” (AAA) Version 16.1. Due to the design of the study dealing with a dosimetric comparison, no comparison in between different algorithms was done. Nevertheless, a recalculation was carried out for the YL‐VMAT method using the “Acuros Algorithm” (Acuros) Version 16.1. The treatment machine used for calculation is a “TrueBeam Vers.2.7” from Varian equipped with a “Millenium 120” MLC model. Yaw tracking was used. If enabled, yaws will automatically move as close as possible to the MLC segments during gantry rotation. An adaption is done dynamically for each segment.^28^ For dose optimization, the “Photon Optimizer” (PO) Version 16.1 from Varian was selected. The prescription dose was set to 40.05 Gy in 15 fractions of 2.67 Gy each and the dose calculation grid size was set to 2.5 mm. Dose normalization was set to the D50% of the PTV. For all 20 patients in total four 6 MV photon treatment plans were created and calculated.

### 2.4 3D‐CRT treatment planning

3D‐CRT plans consisted of two tangential arranged beams as well as field in‐field sub beams. Additionally, in some cases one slightly rotated optional beam to prevent overdosage in the center direction was used. Gantry angles were set patient‐individual to keep the included part of the lung as low as possible. Collimator rotation was set unequal to 0 in between ± 10 degrees.

### 2.5 IMRT treatment planning

Treatment plans for IMRT planning were based on an almost tangential beam setup, similar to 3D‐CRT, with two additional two beams rotated by approximately 16 degrees (range 6–23.2) towards the inner direction. Collimator rotation was also set unequal to 0 in between ± 10 degrees. Dose objectives for the inverse optimization process were defined for the PTV, the left and right lung, the heart, the LAD (left anterior descending) artery, and the external structure. This set of constraints was individually adapted for each patient and is equal within the three inverse planning methods to assure a better comparability.

### 2.6 VMAT treatment planning

The standardized VMAT treatment plans always consisted of two arc beams with approximately 250–260 degrees arc length. The collimator settings were set to 10 and 350 degrees. The field‐size was adapted to the size of the PTV. Also, for YL_VMAT, two control points per degree were set.

### 2.7 YL_VMAT treatment planning

**FIGURE 1:**
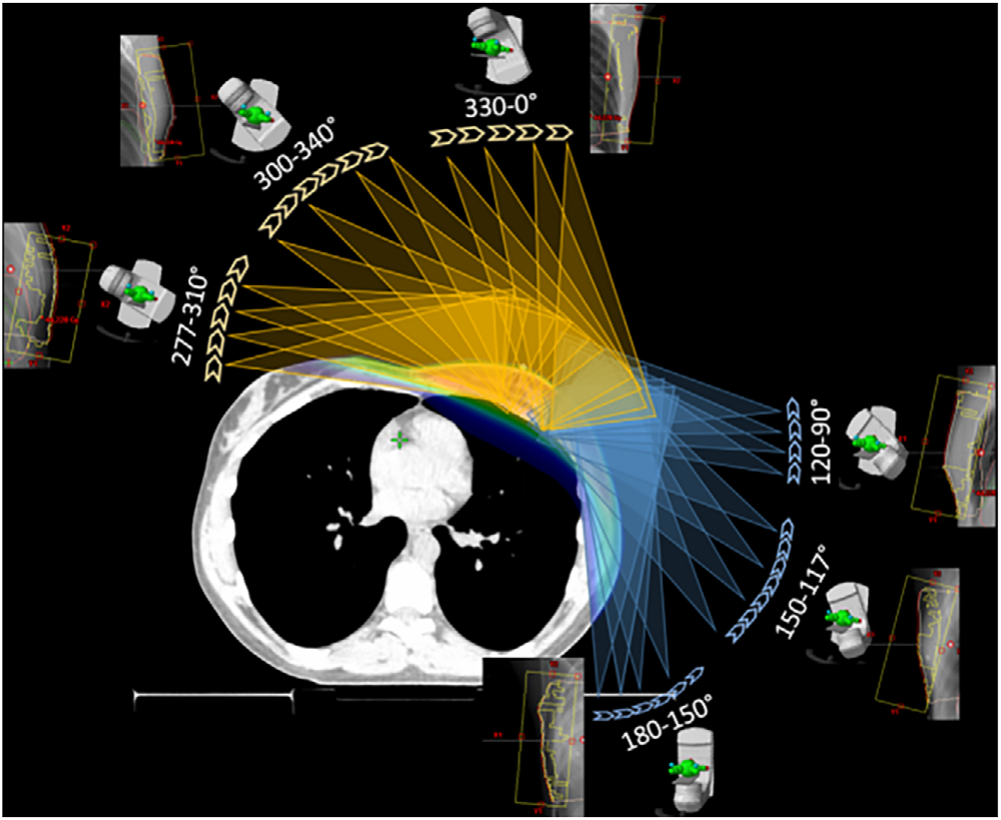
Beam setup for the YL_VMAT technique in detail. Arc segments length is approximately 30 degrees. For each partial arc, collimator rotation is adapted. Frontal breast irradiation is restricted by avoiding arc segments from these directions. YL_VMAT, yaw‐limited volumetric modulated arc therapy.

For the yaw‐limited VMAT planning method a standard plan template was used. This includes six partial VMAT beams with different start, stop and collimator angles, ranging from 180° to 90° and from 0° to 275°, see Figure 1. Depending on the achieved dose distribution, one or more redundant extra beams have been added within already existing rotation sections but with different collimator and yaw settings to saturate the dose in specific regions. If necessary, slight adaption of the start and stop angles have also been performed. The isocenter was placed in the left lung close to the thoracic wall. The yaw, overlapping with the left lung and the heart, was manually limited and shifted towards the PTV outline contour for the first start segments to diminish radiation due to scatter, MLC leakage, and transmission. This results in intended uncovered parts of the PTV within each rotation (please see Supplementary Materials: Figure S1). This study evaluates dose differences within the four planning techniques on rigid CT‐volumes. Therefore, recommended artificial superflab extensions for the planning process to increase the robustness have not been added.

The focus for the inverse planning process was mainly on the dose coverage of the PTV, and low mean doses for the left lung and the heart including the LAD artery.

### 2.8 Evaluation

For the adjacent OARs heart, left lung, and the coronary artery plus the right lung, right breast and the PTV, the outcome for the DVH's have been compared for the different treatment methods. Mean doses and for the left lung different volume characteristics like the V5, V10, and V20 Gy (volume in percent receiving a dose of 5, 10, and 20 Gy) were compared and evaluated. Group 1 contains 10 patients using a DIBH protocol and Group 2 contains 10 patients without breathing instructions (FB). These two groups have also been compared with each other.

### 2.9 Statistical analysis

To evaluate the statistical significance of OAR dose sparing using the YL‐VMAT planning method, Analysis of variance (ANOVA) tests were performed. *P*‐values have been calculated in pairwise analyzes between each YL‐VMAT and the three other techniques. A *p*‐value < 0.05 was assumed to be statistically significant.

## 3 RESULTS

**FIGURE 2:**
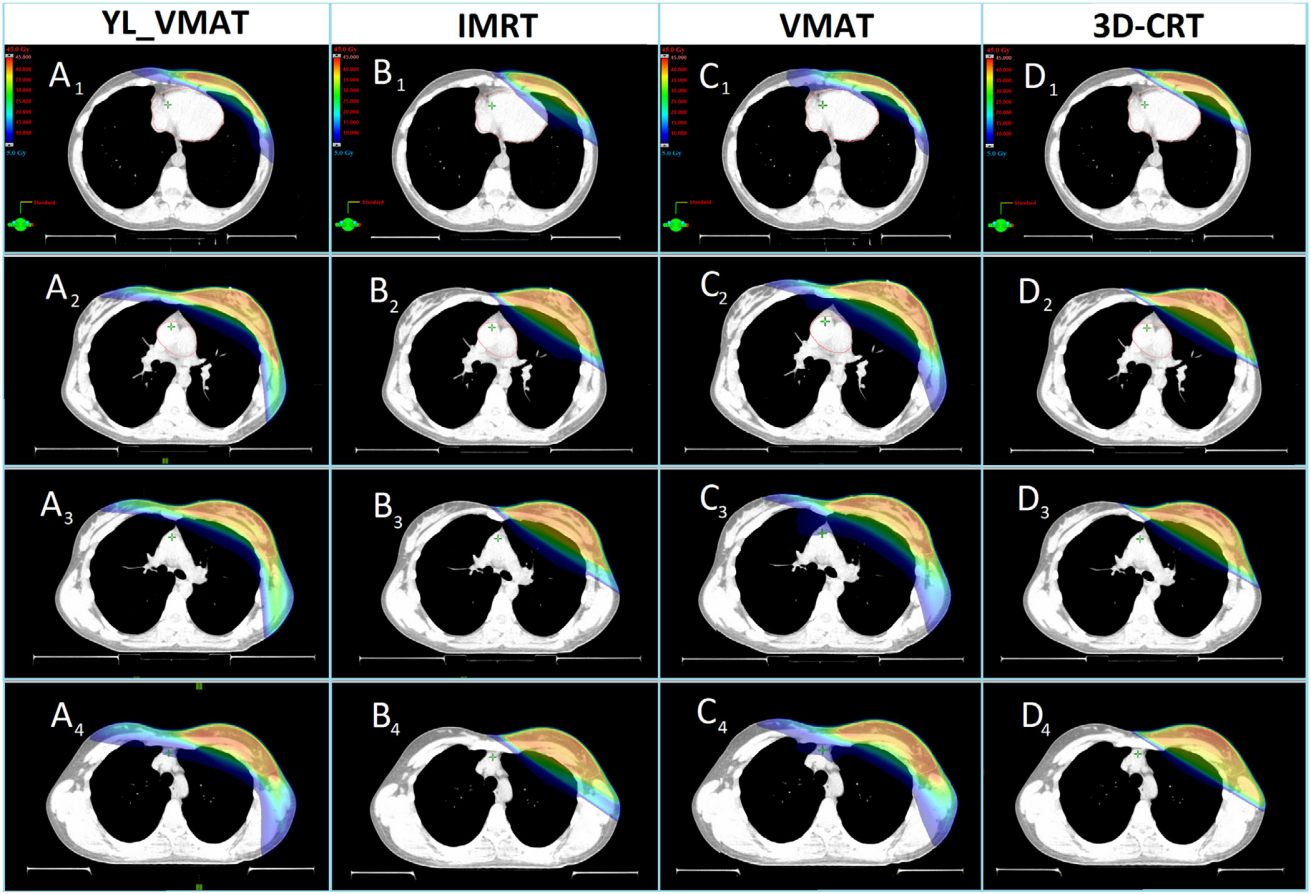
Transversal dose distributions for four different regions of the left breast (PTV) with a lower dose threshold of 5 Gy (12.5%) for YL_VMAT, IMRT, VMAT, and 3D‐CRT, respectively. Representative parts of the thorax are selected: A1: Caudal part of the PTV including the heart; A2: More cranial part with heart upper region; A3: Middle part of the PTV; A4: Cranial part of the PTV. IMRT, intensity modulated radiation therapy; PTV, planning target volume; VMAT, volumetric modulated arc therapy; YL_VMAT, yaw‐limited volumetric modulated arc therapy; 3D‐CRT, 3D‐conformal radiotherapy.

Four transverse slices give a general overview of the region of the left breast, including the resulting dose distributions for the four different irradiation techniques arranged for comparison, as shown in Figure 2. The mean monitor units for both groups are 1144, 1135, 700, and 301 for YL_VMAT, IMRT, VMAT, and 3D‐CRT, respectively.

**FIGURE 3:**
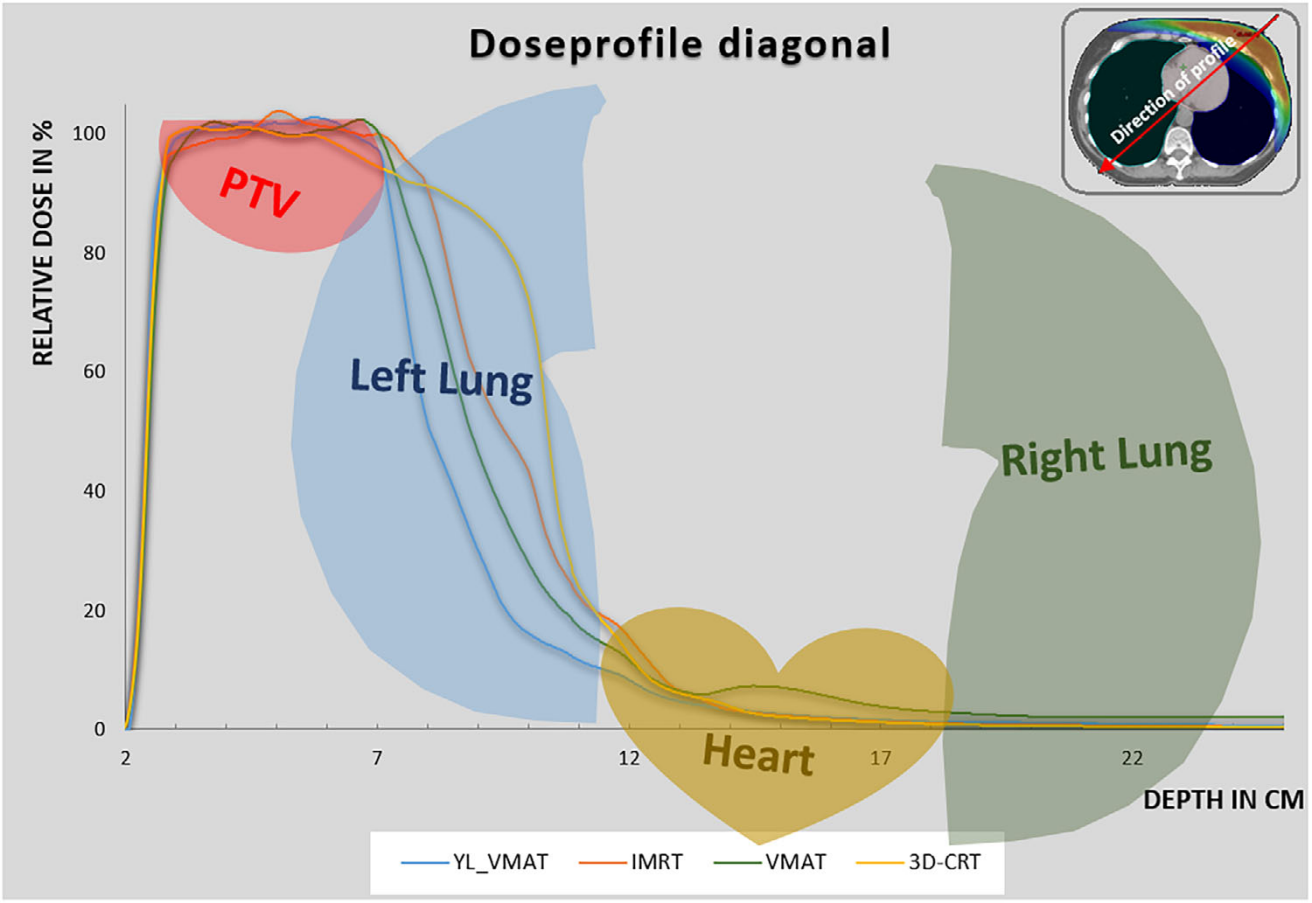
Dose fall‐off from the PTV towards the chest wall shown as a dose‐profile. Blue, orange, green, and yellow solid lines are for YL_VMAT, IMRT, VMAT, and 3D‐CRT, respectively. For better illustration the organs are projected and roughly correlate with presented depth. IMRT, intensity modulated radiation therapy; PTV, planning target volume; VMAT, volumetric modulated arc therapy; YL_VMAT, yaw‐limited volumetric modulated arc therapy; 3D‐CRT, 3D‐conformal radiotherapy.

The dose fall‐off in the diagonal direction starting at the chest surface to the patient's center as illustrated in Figure 3 for one selected patient. For the YL_VMAT technique, the dose fall‐off runs most steeply. The profile length from 95% down to 50% of the dose is about 9 mm. For IMRT, it is 19 mm, for VMAT it is about 16 mm, and for 3D‐CRT it is about 35 mm. For the VMAT technique, the dose gradient is also better compared to IMRT and 3DCRT but results in an increased mean dose to the contralateral lung.

**FIGURE 4:**
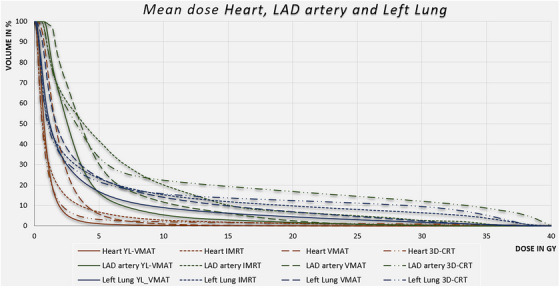
Dose volume histogram comparison between YL_VMAT (solid lines), IMRT (dotted lines), VMAT (dashed lines) and 3D‐CRT (broken lines) for the heart (brown), the coronary artery (green), and the left lung (blue). DVH is representing mean values over 10 patients for DIBH. The considerably dose reduction for the organs with YL_VMAT is obvious. DIBH, deep inspiration breath‐hold; IMRT, intensity modulated radiation therapy; YL_VMAT, yaw‐limited volumetric modulated arc therapy; 3D‐CRT, 3D‐conformal radiotherapy.

For the adjacent OARs affected by radiation, Table 1 gives an overview of dose accumulations for the DIBH and the FB protocol (please see also a comparison between AAA and Acuros algorithm in Supplementary Materials: Table S1). For the heart, the LAD artery, and the left lung, dose exposures are considerably lower with the YL_VMAT technique. For Group 1, this is also shown in a dose volume histogram in Figure 4. In Group 1 (DIBH), the comparison between YL‐VMAT and standard techniques IMRT, VMAT, and 3D‐CRT showed that YL‐VMAT achieved lower mean doses for the heart (1.05 Gy vs. 1.73 Gy, 2.16 Gy and 1.44 Gy), the LAD artery (3.68 Gy vs. 6.53 Gy, 5.13 Gy and 8.64 Gy) and the left lung (3.59 Gy vs. 5.39 Gy, 4.79 Gy and 5.87 Gy), respectively (the corresponding *p*‐values are presented in Table 1). In Group 2 (FB), the corresponding mean doses for the left lung and cardiac structures were also lower with the YL‐VMAT method than with IMRT (heart: 1.70 Gy vs. 2.44 Gy; LAD: 6.50 Gy vs. 11.97 Gy; left lung: 3.10 Gy vs. 4.72 Gy), VMAT (heart: 1.70 Gy vs. 2.52 Gy; LAD: 6.50 Gy vs. 9.06 Gy; left lung: 3.10 Gy vs. 4.46 Gy) and 3D‐CRT technique (heart: 1.70 Gy vs. 2.78 Gy; LAD: 6.50 Gy vs. 15.09 Gy; left lung: 3.10 Gy vs. 5.77 Gy); please see Table 1 for the corresponding *p*‐values. The advantage of the DIBH method lies in the dose‐sparing effect on the heart and, in particular, on the LAD artery, which runs along the anterior wall of the heart between the left and right ventricles in topographical proximity to the left thoracic wall.

**TABLE 1:** Dosimetric comparison between YL_VMAT, IMRT, VMAT, and 3D‐CRT.

DIBH (G1)	YL_VMAT	IMRT	p‐value	VMAT	p‐value	3D‐CRT	p‐value
Heart mean dose in Gy	1.05 ± 0.27	1.73 ± 1.19	0.116	2.16 ± 0.63	0.001	1.44 ± 1.00	0.280
LAD artery mean dose in Gy	3.68 ± 2.02	6.53 ± 3.50	0.049	5.13 ± 2.12	0.155	8.64 ± 7.41	0.069
LAD artery V15 in %	2.98 ± 7.71	10.19 ± 12.63	0.161	6.20 ± 8.97	0.425	19.33 ± 22.10	0.051
Left lung V5 in %	16.83 ± 1.69	23.28 ± 4.23	0.001	23.76 ± 2.59	0.001	21.64 ± 4.16	0.005
Left lung V10 in %	9.00 ± 1.80	15.27 ± 3.10	0.001	14.02 ± 1.53	0.001	15.71 ± 3.39	0.001
Left lung V20 in %	4.49 ± 1.51	9.82 ± 2.28	0.001	6.96 ± 0.97	0.001	12.43 ± 3.01	0.001
Left lung mean in Gy	3.59 ± 0.41	5.39 ± 0.88	0.001	4.79 ± 0.55	0.001	5.87 ± 1.16	0.001
Right lung mean in Gy	0.28 ± 0.12	0.14 ± 0.08	0.008	1.43 ± 0.30	0.009	0.09 ± 0 04	0.001
Right breast mean in Gy	4.46 ± 1.92	0.85 ± 0.59	0.001	3.44 ± 0.81	0.001	0.43 ± 0.15	0.001

FB (G2)	YL_VMAT	IMRT	*p*‐value	VMAT	*p*‐value	3D‐CRT	*p*‐value
Heart mean dose in Gy	1.70 ± 0.81	2.44 ± 0.87	0.080	2.52 ± 0.52	0.020	2.78 ± 1.79	0.116
LAD artery mean dose in Gy	6.50 ± 3.73	11.97 ± 6.16	0.035	9.06 ± 4.65	0.214	15.09 ± 7.92	0.009
LAD artery V15 in %	13.59 ± 13.43	29.46 ± 17.53	0.045	22.67 ± 19.39	0.263	37.22 ± 22.00	0.132
Left lung V5 in %	15.01 ± 3.24	19.58 ± 3.90	0.015	21.21 ± 3.99	0.002	20.99 ± 6.40	0.022
Left lung V10 in %	7.27 ± 2.21	12.52 ± 3.26	0.001	12.59 ± 2.86	0.001	15.35 ± 5.36	0.001
Left lung V20 in %	2.83 ± 1.06	8.31 ± 2.28	0.001	6.07 ± 1.79	0.001	12.03 ± 4.78	0.001
Left lung mean in Gy	3.10 ± 0.52	4.72 ± 0.90	0.001	4.46 ± 0.69	0.001	5.77 ± 1.86	0.001
Right lung mean in Gy	0.45 ± 0.37	0.14 ± 0.06	0.022	1.43 ± 0.54	0.001	0.07 ± 0.06	0.007
Right breast mean in Gy	6.33 ± 3.69	0.71 ± 0.53	0.001	3.59 ± 1.45	0.053	0.39 ± 0.33	0.001

Abbreviations: 3D‐CRT, 3D‐conformal radiotherapy; IMRT, intensity modulated radiation therapy; *p*‐value, significance value performed via “Analysis of variance” (ANOVA); values right to “ ± ” are SD, standard deviation; YL_VMAT, yaw‐limited volumetric modulated arc therapy.

Nevertheless, in this study, DIBH leads to an increased mean dose for the ipsilateral lung, regardless of the treatment technique. For example, for the YL‐VMAT technique, the mean dose to the left lung is even significantly lower with 3.10 Gy for FB versus 3.59 Gy (*p* = 0.037) for DIBH, respectively.

For Groups 1 and 2, the volume of the left lung exposed by 5, 10, and 20 Gy was significantly lower with the YL‐VMAT technique than with all other standard techniques. Regarding the V15 Gy of the LAD artery, YL_VMAT is also superior. Details are also given in Table 1.

Due to contralateral irradiation directions, the mean dose for the right breast is increased considerably for both VMAT techniques. For Group 1 mean doses are 4.46 Gy and 3.44 Gy and for Group 2 6.33 Gy and 3.59 Gy for YL‐VMAT and VMAT, respectively. Compared to IMRT and 3D‐CRT, mean doses for the YL_VMAT technique are even significantly increased (*p* < 0.05).

**FIGURE 5:**
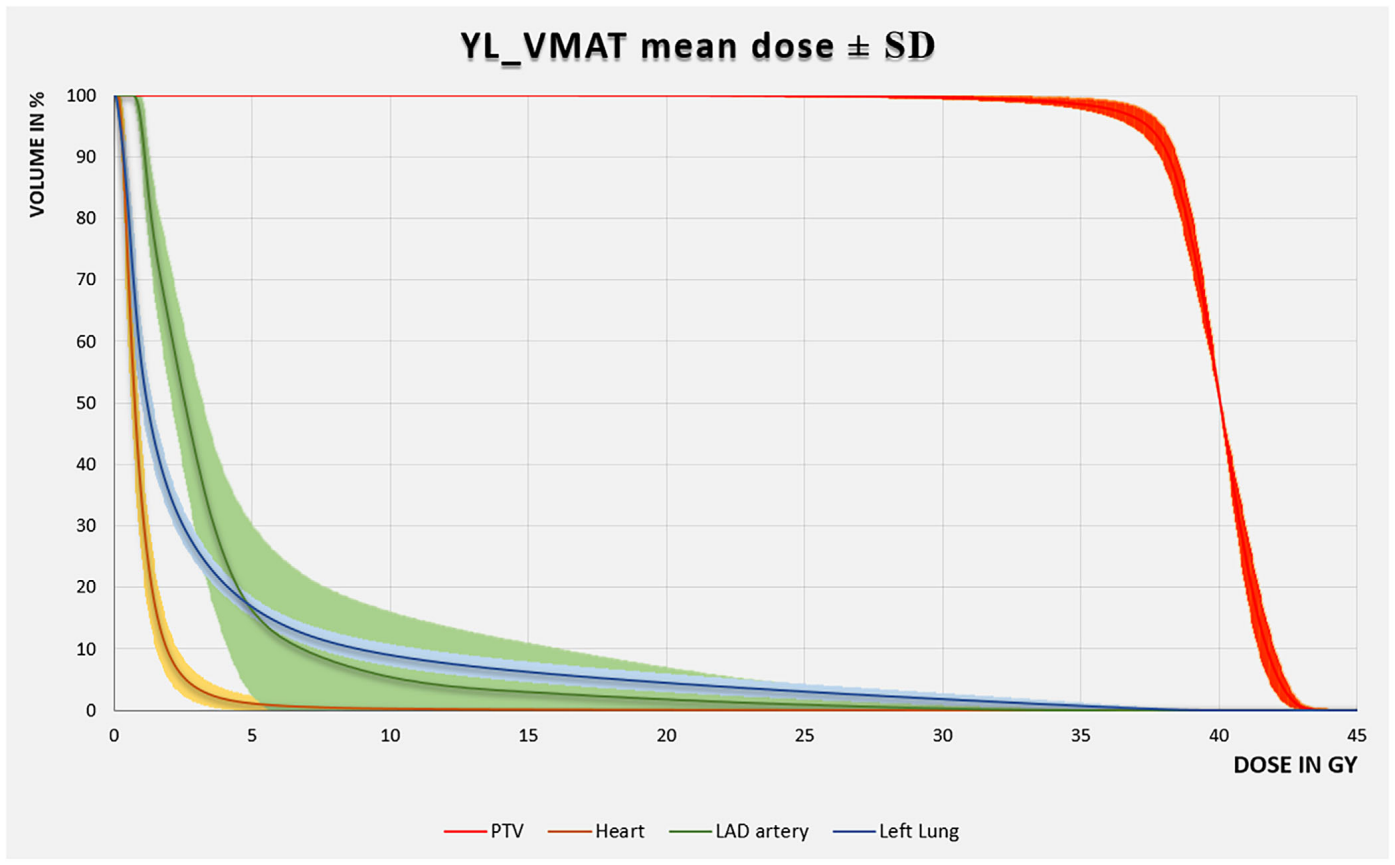
Dose volume histogram for Group 1 (DIBH) with YL_VMAT technique for the PTV (red lines), the heart (brown lines), the coronary artery (green lines), and the left lung (blue lines). The variation of the mean dose values are shown via the standard deviation (SD) and are most pronounced for the LAD artery. DIBH, deep inspiration breath‐hold; PTV, planning target volume; YL_VMAT, yaw‐limited volumetric modulated arc therapy.

For the 10 patients in Group 1, a mean DVH including the standard deviations is shown in Figure 5. Due to patients’ anatomy, the width of the standard deviation is increased for the LAD artery.

## 4 DISCUSSION

This study investigated dose exposures to adjacent OARs during irradiation therapy for left‐sided breast cancer patients using four different planning approaches. The multi‐field YL_VMAT technique with several partial, yaw‐limited arc segments is able to reduce mean doses to the OARs considerably. In addition to selecting the optimal irradiation directions for the treatment field setup, minimizing the dose contributions induced by MLC leaf transmission and inter‐leaf leakage are technical opportunities for OAR dose sparing. For the Varian Millennium 120 MLC, the average transmission is 2% and the maximal inter‐leaf leakage is about 2.5%.^29^ Thus, limiting the yaw‐field size in the direction of the MLC movement directly affects the dose exposure, resulting in a steeper and more pronounced dose fall‐off from the thoracic wall to the ipsilateral lung. Nevertheless, the narrow field design results in an increased number of monitor units for the YL_VMAT technique by a factor of 1.1, 1.8, and 4.0 compared to IMRT, VMAT, and 3D‐CRT, respectively. This corresponds also with an extended beam on time. Additionally, using a split beam technique usually increases the number of treatment fields. On the other hand, a split beam setup has advantages when using a DIBH protocol as it reduces beam on time per field.^10^, ^30^

Considering patient positioning errors and anatomical changes during radiation therapy, the use of virtual bolus structures is recommended for optimization with VMAT treatment methods.^31^ Thus, the treatment planning process is more time‐consuming for YL‐VMAT and VMAT, compared to IMRT and 3D‐CRT. Especially, the patient individually yaw adjustments for YL_VMAT, lead to a higher effort. However, the plan robustness for VMAT planning methods is as safe or better than IMRT and 3D‐CRT for localization errors.^32^, ^33^, ^34^

Regarding the OARs, long‐term mortality studies revealed an increased risk for developing subsequent lung cancer after radiation therapy for breast cancer.^35^, ^36^ Radiation‐induced lung cancer is an important risk factor, leading to recommendations to use modern treatment planning methods like IMRT.^37^

Kubo et al. studied the impact of the extension of the lung tissue located within the tangential treatment fields more closely.^38^ As a central result, this study showed that distances of more than 1.8 cm from the thoracic wall to the lung or the lung volume within the radiation field are a significant risk factor for radiation‐induced bronchiolitis obliterans organizing pneumonia (BOOP). With the YL_VMAT technique, the V20, V10, and the V5 Gy of the left lung as well as the mean dose, are significantly lower compared to the other demonstrated techniques. Especially for 3D‐CRT but also for IMRT, the volume receiving higher doses is considerably increased due to the tangential arrangement of the treatment fields. Hence, the distance from the thoracic wall to the high‐dose‐isodose line is often larger than 1.8 cm. For the conventional VMAT technique, diminishing the dose to 10 Gy or less was also possible. In general, higher exposures to the whole lung increase lung pneumonitis risk, as well as the risk of developing lung cancer after radiation therapy.^39^ Therefore, minimizing mean doses could be beneficial in reducing these risks.

Regarding the dose exposure to the heart, in the first 9 years after radiotherapy, the cumulative incidence of acute coronary events (ACE) associated with irradiation of the whole heart, is increased by 16.5% per Gy.^40^ Data published based on a collective of 910 female patients receiving radiation therapy resulted in a mean heart dose of 2.37 Gy (range, 0.51–15.25 Gy).^40^ The German society of radiation oncology (DEGRO, Deutsche Gesellschaft für Radioonkologie) recommends keeping the mean heart dose below 2.5 Gy.^41^ Other studies reported an increased cardiac mortality ratio for female breast cancer patients treated with radiation.^35^, ^40^, ^42^ Milo et al. also published a 10‐year cumulative risk of cardiac events of about 2.1% Especially by using modern radiotherapy techniques and DIBH, a decreased heart mean dose compared to FB is achievable.^16^, ^18^, ^20^ Our results also show a better dose‐sparing effect with the DIBH setup. Anyway, with the yaw optimized technique, even for FB setup, the mean heart dose has been further reduced well below the limit of 2.5 Gy.

Regarding the LAD artery dose‐sparing capabilities, the YL_VMAT technique leads to lower mean values as well. In particular, for Group 2 patients, it is possible to reduce the dose to the LAD artery much better than with IMRT, VMAT, and 3D‐CRT. Therefore, patients being non‐compliant or treated at radiation therapy sites without respiratory management devices could benefit from the use of the YL_VMAT technique. An odds ratio (OD) of about 1.24 (95% CI 0.52–2.95) for mean doses from 5 to 20 Gy was observed with an increased need for coronary intervention. It is therefore recommended to keep the dose to the coronary artery as low as possible in order to reduce the risk of subsequent radiation‐induced vasoconstriction.^43^ Taylor et al. found a strong association of higher radiation doses with frequent injury for the coronary artery.^44^ DEGRO cancer expert panel recommends a mean dose of less than 10 Gy for the LAD artery.^41^ In a reanalysis of RTOG 0617, McKenzie et al. found an association of the LAD artery V15 Gy ≥ 10% with the risk of all‐cause mortality.^45^ Overall, the V15 Gy of the LAD artery is minimized by the YL_VMAT technique, both for DIBH as for the FB protocol. However, for DIBH the volume is further decreased compared to FB, and only for YL_VMAT and VMAT, values below 10% were achieved.

However, the disadvantage of VMAT radiation techniques for patients with left breast cancer is that they often lead to a higher dose exposure of the contralateral breast.^46^, ^47^ Furthermore, especially split field designs can lead to an increased dose to the contralateral breast.^7^

Stovall et al. analyzed patient's age with respect to accumulated mean dose to the contralateral breast.^48^ For doses higher than 1 Gy and women younger than 40 years of age, they found a 2.5‐fold greater risk for contralateral breast cancer compared to unexposed women. In contrast, for women older than 40 years no increased risk was observed. Therefore, in particular, for younger women, disadvantages by higher dose exposures of the contralateral breast have to be weighed up against the advantages in dose sparing for the heart and the lungs. By adapting dose constraints during the inverse planning process or limiting irradiation directions, dose exposures to OARs can be “shifted” among each other for all four planning techniques. Nevertheless, especially the adjustment of start and stop angles for the YL_VMAT and VMAT techniques has an important impact.

Regarding the overall risk of developing radiation‐induced second cancer, there is a growing concern of increasing cases of second malignancies due to more cancer survivor patients. About 17%–19% of cancer survivors develop a second malignancy, with about 5% of all cases being radiation‐induced.^49^, ^50^ From 154.697 women who received radiation therapy, 13% developed a secondary malignancy, and within these, approximately 3.4% were radiation‐induced. The authors, therefore, concluded that breast cancer patients were at increased risk of secondary malignancies, regardless of whether or not they had received radiotherapy.^51^ Additionally, another issue of concern is the use of VMAT techniques, which may lead to an increased rate of secondary malignancies mainly through an increased low‐dose bath.^52^, ^53^ Depending on how the VMAT technique is applied, similar risks for the induction of secondary cancer as with 3D‐CRT were identified.^52^, ^54^ Furthermore, second malignancies often occur in regions of high doses within the 3D‐CRT treatment fields.^55^ Taking into account the advantages in dose‐sparing capabilities for the heart, the LAD artery and the left lung, YL_VMAT is a promising technique to further improve the outcome of patients with left‐sided breast cancer.

However, this study has several limitations. One limitation of the study is its retrospective design, which creates a classical dosimetric study by comparing different treatment methods. Furthermore, this is only a single‐center study. Delineations made by Mvision lead to better comparability but may not be accurate in each case. Nevertheless, it has to be mentioned, that contouring by AI‐based algorithms results in high‐quality structures.^56^ Another issue is the fact that the shown YL_VMAT technique is not fully standardized. Due to different anatomies, only the initial setup is template‐based. Individual adaptions have to be made for a better outcome, including yaw positions, collimator rotations, and minimal modifications for arc start and stop angles. The presented setup was also not applicable to all patients with left‐sided breast cancer. Some modifications have to be made for application of the YL‐VMAT, resulting in a semi, non‐standardized approach. This study showed that the YL‐VMAT approach is suitable for relatively small PTV volumes, respectively, small breasts. Further investigations may also lead to an adapted setup for larger PTV volumes.

## 5 CONCLUSION

A yaw‐limited VMAT method (YL_VMAT) for left‐sided breast cancer patients results in a high dose fall‐off to the thoracic wall. Dose exposures to radiosensitive organs like the heart, the LAD artery, and the left lung can be reduced considerably to values well below known critical thresholds. Overall, in comparison to IMRT, conventional VMAT, and 3D‐CRT, substantial dose sparing to the OARs has been achieved.

This study also confirms well‐known heart dose‐sparing capabilities using a DIBH protocol instead of FB. Nevertheless, the results for FB are on a comparable level.

All dose‐sparing advantages for adjacent OARs must be carefully balanced against an increased mean dose for the contralateral breast. Patient individual decisions and focus are highly recommended.

The demonstrated treatment planning method is also applicable for chest wall irradiations and benefits in dose sparing for OARs would be even higher. Further investigations have to be made for a wider scope of application and automation.

## AUTHOR CONTRIBUTIONS

Gerhard Pollul (Physicist) is responsible for the retrospective analysis, statistical evaluation, data collection, and interpretation of the results. All listed co‐authors contributed to the style of writing in all sections, revisions, and figures selection.

## CONFLICT OF INTEREST STATEMENT

The authors declare no conflicts of interest.

## Supporting information



Supporting Information

Supporting Information

Supporting Information
