# Supplementary material for: Improving organ dose sparing in left‐sided breast cancer with yaw‐limited volumetric modulated arc therapy: A dosimetric comparison to conventional and intensity modulated radiation therapy approaches
Source: J Appl Clin Med Phys. 2025 Feb 28;26(5):e70041. doi: 10.1002/acm2.70041 (PMC12059266; doi:10.1002/acm2.70041)
Supplement: Supplementary file 1 — Supporting Information [file ACM2-26-e70041-s003.docx]

**Improving organ dose sparing in left-sided breast cancer with yaw‑limited Volumetric Modulated Arc Therapy: A dosimetric comparison to conventional and intensity modulated radiation therapy approaches**

Gerhard Pollul, Sascha Grossmann, Heiko Karle, Tilman Bostel and Heinz Schmidberger

Gerhard Pollul

Universitatsmedizin der Johannes Gutenberg-Universitat Mainz

Department of Radiation Oncology

Langenbeckstraße 1

55131 Mainz

Germany

Email: [gerhard.pollul@unimedizin-mainz.de](mailto:gerhard.pollul@unimedizin-mainz.de)

**Improving organ dose sparing in left-sided breast cancer with yaw‑limited Volumetric Modulated Arc Therapy**

Sascha Grossmann

Universitatsmedizin der Johannes Gutenberg-Universitat Mainz

Department of Radiation Oncology

Langenbeckstraße 1

55131 Mainz

Germany

Email: [sascha.grossmann@unimedizin-mainz.de](mailto:sascha.grossmann@unimedizin-mainz.de)

Heiko Karle

Universitatsmedizin der Johannes Gutenberg-Universitat Mainz

Department of Radiation Oncology

Langenbeckstraße 1

55131 Mainz

Germany

Email: heiko.karle@unimedizin-mainz.de

Tilman Bostel

Universitatsmedizin der Johannes Gutenberg-Universitat Mainz

Department of Radiation Oncology

Langenbeckstraße 1

55131 Mainz

Germany

Email: tilman.bostel@unimedizin-mainz.de

Heinz Schmidberger

Universitatsmedizin der Johannes Gutenberg-Universitat Mainz

Department of Radiation Oncology

Langenbeckstraße 1

55131 Mainz

Germany

Email: [heinz.schmidberger@unimedizin-mainz.de](mailto:heinz.schmidberger@unimedizin-mainz.de)
