# Supplementary material for: Improving organ dose sparing in left‐sided breast cancer with yaw‐limited volumetric modulated arc therapy: A dosimetric comparison to conventional and intensity modulated radiation therapy approaches
Source: J Appl Clin Med Phys. 2025 Feb 28;26(5):e70041. doi: 10.1002/acm2.70041 (PMC12059266; doi:10.1002/acm2.70041)
Supplement: Supplementary file 2 — Supporting Information [file ACM2-26-e70041-s001.docx]

**Supplementary Materials:**

**Figure S1:** Detailed beam setup and optimization constraints for the YL_VMAT technique.


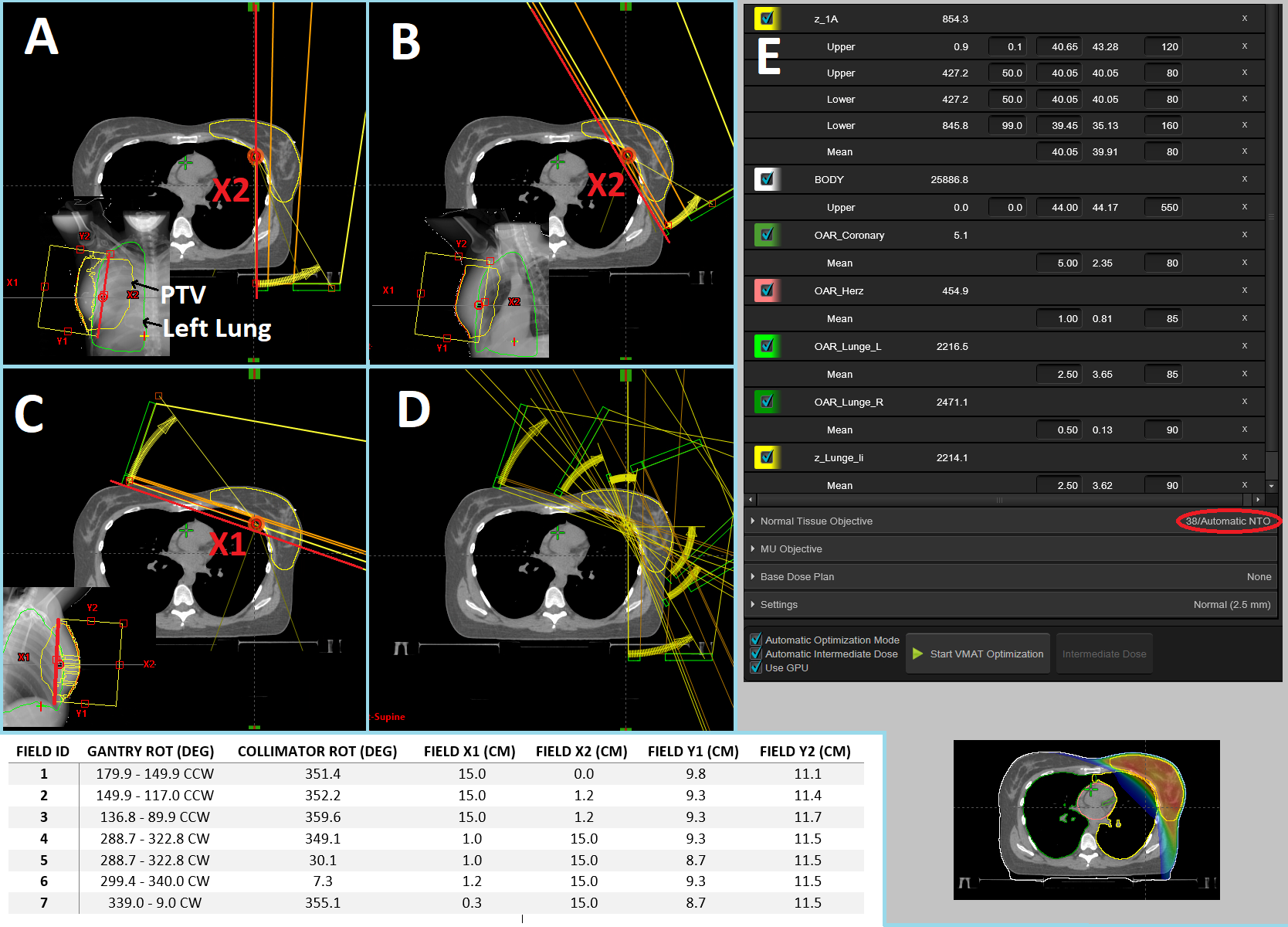


**Figure S1**. A: First partial arc segment (ID 1) with 30 degrees arc length and with narrow field size in x‑direction. B: Second partial arc segment (ID 2) with 32 degrees arc length and PTV-extend adapted yaw-position. C: Forth arc segment (ID 4) with 34.1 degrees arc length. D: All partial arcs segments used for this patient (ID 1 – ID 7). All images show projections each for the start angle. E: Dose constraints used for optimization. NTO was set to a lower level.

**Abbreviations:** YL_VMAT = Yaw-limited Volumetric modulated arc therapy; OAR_Lunge_L = Left part of the lung; OAR_Lunge_R = Right part of the lung; z_Lunge:li = Left part of the lung cropped by 3 mm to the PTV (left breast); z_1A = PTV (left breast) cropped by 3 mm to the skin.
