# Supplementary material for: Improving organ dose sparing in left‐sided breast cancer with yaw‐limited volumetric modulated arc therapy: A dosimetric comparison to conventional and intensity modulated radiation therapy approaches
Source: J Appl Clin Med Phys. 2025 Feb 28;26(5):e70041. doi: 10.1002/acm2.70041 (PMC12059266; doi:10.1002/acm2.70041)
Supplement: Supplementary file 3 — Supporting Information [file ACM2-26-e70041-s002.docx]

**Supplementary Materials:**

**Table S1:** Dosimetric comparison for YL_VMAT, calculated with AAA and Acuros.

| **DIBH (G1)** | **YL_VMAT (AAA)** | **YL_VMAT (Acuros)** | **Δ (AAA-Acuros)** |
| --- | --- | --- | --- |
| Heart mean dose in Gy | 1.05 ± 0.27 | 0.87 ± 0.25 | 0.18 |
| LAD artery mean dose in Gy | 3.68 ± 2.02 | 3.05 ± 2.11 | 0.63 |
| LAD artery V15 in % | 2.98 ± 7.71 | 2.98 ± 7.82 | 0.00 |
| Left lung V5 in % | 16.83 ± 1.69 | 16.54 ± 1.71 | 0.29 |
| Left lung V10 in % | 9.00 ± 1.80 | 9.48 ± 1.55 | -0.48 |
| Left lung V20 in % | 4.49 ± 1.51 | 4.48 ± 1.55 | 0.01 |
| Left lung mean in Gy | 3.59 ± 0.41 | 3,33 ± 0.43 | 0.26 |
| Right lung mean in Gy | 0.28 ± 0.12 | 0.34 ± 0.10 | -0.06 |
| Right breast mean in Gy | 4.46 ± 1.92 | 4.70 ± 1.91 | -0.24 |
| **FB (G2)** | ***YL_VMAT (AAA)*** | ***YL_VMAT (Acuros)*** | ***YL_VMAT (Acuros)*** |
| Heart mean dose in Gy | 1.70 ± 0.81 | 1.18 ± 0.41 | 0.52 |
| LAD artery mean dose in Gy | 6.50 ± 3.73 | 6.04 ± 3.73 | 0.46 |
| LAD artery V15 in % | 13.59 ± 13.43 | 13.35 ± 13.35 | 0.24 |
| Left lung V5 in % | 15.01 ± 3.24 | 13.71 ± 3.23 | 1.30 |
| Left lung V10 in % | 7.27 ± 2.21 | 7.41 ± 2.17 | -0.14 |
| Left lung V20 in % | 2.83 ± 1.06 | 3.00 ± 1.15 | -0.17 |
| Left lung mean in Gy | 3.10 ± 0.52 | 2.77 ± 0.50 | 0.33 |
| Right lung mean in Gy | 0.45 ± 0.37 | 0.51 ± 0.31 | -0.06 |
| Right breast mean in Gy | 6.33 ± 3.69 | 6.56 ± 3.70 | -0.23 |

**Abbreviations:** YL_VMAT = Yaw-limited Volumetric modulated arc therapy; Δ = Difference; values right to “±” are SD = Standard Deviation.
